# Heterodisomy in the *GNAS* locus is also a cause of pseudohypoparathyroidism type 1B (iPPSD3)

**DOI:** 10.3389/fendo.2024.1505244

**Published:** 2024-12-16

**Authors:** Africa Manero-Azua, Yerai Vado, Judith Gonzàlez Morlà, Eduard Mogas, Arrate Pereda, Guiomar Perez de Nanclares

**Affiliations:** ^1^ Rare Disease Research Group, Molecular (Epi) Genetics Laboratory, Bioaraba Health Research Institute, Araba University Hospital, Vitoria-Gasteiz, Spain; ^2^ Pediatric Endocrinology Section, Hospital Comarcal de Palamós, Girona, Spain; ^3^ Pediatric Endocrinology Section, Hospital Universitari Vall d’Hebron, Barcelona, Spain

**Keywords:** PHP1B, iPPSD3, *GNAS*, heterodisomy, upd(20)pat, MS-MLPA

## Abstract

**Objective:**

To identify the genetic cause underlying the methylation defect in a patient with clinical suspicion of PHP1B/iPPSD3.

**Design:**

Imprinting is an epigenetic mechanism that allows the regulation of gene expression. The *GNAS* locus is one of the loci within the genome that is imprinted. When the methylation pattern is affected, it causes pseudohypoparathyroidism type 1B (PHP1B) or inactivating PTH/PTHrP signaling disorder 3 (iPPSD3). Paternal uniparental isodisomy (iUPDpat) of the chromosomal region comprising the *GNAS* locus has been described as one of the possible underlying genetic causes of the methylation alteration.

**Methods:**

We present the case of a patient clinically diagnosed with iPPSD3. We performed a commercial methylation-specific multiplex ligation-dependent probe amplification (MS-MLPA), single-nucleotide polymorphism (SNP) array, and microsatellite study. In addition, we designed a custom MS-MLPA to analyze *GNAS* and nearby differentially methylated regions (DMRs).

**Results:**

A methylation defect at the four *GNAS*-DMRs was detected, confirming the clinical diagnosis. Complementary techniques revealed the presence of a mixed isodisomy and heterodisomy of chromosome 20. Surprisingly, the *GNAS* locus was located on the heterodisomic zone.

**Conclusions:**

Paternal uniparental heterodisomy (hUPD) at the *GNAS* locus is also a genetic defect associated with iPPSD3. In the absence of parental samples, our custom MS-MLPA allows for the detection of a methylation defect at the *GNAS* locus and flanking DMRs, suggestive of uniparental disomy (UPD). We also suggest updating the actual guidelines to include hUPD at the *GNAS* locus as a cause of iPPSD3.

## 1 Introduction

The *GNAS* locus is an imprinted locus located in the long arm of chromosome 20 (20q13.2-20q13.3) (1). Due to its complex imprinted pattern, the expression of the multiple transcripts derived from the locus is dependent on the parental origin (2).

There are four first alternative exons, which are spliced onto exons 2-13, that are common for all the transcripts. These alternative first exons are regulated by four differentially methylated regions (DMRs), which are *cis*-acting elements that control the parent-of-origin expression: *GNAS-NESP:*TSS-DMR, *GNAS-AS1:*TSS-DMR, *GNAS-XL:*Ex1-DMR and *GNAS A/B:*TSS-DMR. The first one is methylated in the paternal allele and in consequence, is expressed from the maternal allele. In contrast, the other three are imprinted in the maternal allele and expressed from the paternal one (3–7).

When the methylation of the complex locus is abnormal in the DMRs, it gives rise to the disease known as pseudohypoparathyroidism type 1B (PHP1B, OMIM#603233) or according to the recent recommendations, inactivating PTH/PTHrP signaling disorder 3 (iPPSD3) (8). The methylation defect can affect one or multiple DMRs and it can be partial or complete (9), but all patients affected with iPPSD3 show loss of methylation (LOM) at the *GNAS A/B:*TSS-DMR (10). Clinically, iPPSD3 patients have been characterized by parathyroid hormone (PTH) resistance, leading to hypocalcemia and hyperphosphatemia. Sometimes, thyroid stimulating hormone (TSH) resistance is also present. In these patients, Albright’s Hereditary Osteodystrophy (AHO) phenotype is commonly absent, and if present, it is usually milder (10, 11). Some additional signs include excessive intrauterine growth (12, 13).

Of the iPPSD3 patients, 15%-20% of the cases are familial, with LOM at the *GNAS A/B:*TSS-DMR secondary to deletion of 3.3 kb in the *STX16* gene. This genetic defect is inherited from the maternal lineage in an autosomal dominant manner (10), and is accompanied by a *GNAS-AS2*:TSS-DMR partial LOM (14). However, there are some rare familial cases with different deletions and duplications affecting only the *GNAS A/B:*TSS-DMR or all the DMRs (10). Apart from the familial cases, there are also sporadic iPPSD3 patients (15). In the majority of the sporadic cases, a methylation alteration usually affects the four *GNAS*-DMRs and the genetic cause of the methylation defect remains unknown. Nevertheless, in approximately 8%-20% of them, paternal uniparental isodisomy (iUPD) including 20q13 has been detected (15–17).

Uniparental disomy (UPD) is an abnormal genetic condition in which the individual carries a pair of chromosomes (or segments of chromosomes) from the same progenitor without a contribution from the other parent. Two different types of UPD can be distinguished: uniparental heterodisomy (hUPD), when both homologous chromosomes are inherited from one parent; and, uniparental isodisomy (iUPD), when a single chromosome is inherited from the parent and it duplicates (18, 19). Although it is very rare, translocations giving rise to paternal uniparental disomy of chromosome 20, upd(20)pat, have also been described (15).

For the detection of paternal iUPD in 20q13 [iUPD(20q)pat], current international guidelines for the diagnosis of PHP and related disorders recommend the analysis of microsatellites or short tandem repeats (STR) in the trio (if the samples are available) and/or single nucleotide polymorphism (SNP) array (15). However, if the samples of the parents are not available, STR analysis can be non-informative (20).

## 2 Materials and methods

### 2.1 Case presentation

We present the clinical case of a 3-year-old boy who was admitted to the hospital emergency department due to a second seizure episode in the context of a viral illness caused by influenza A. The initial blood tests in the emergency room revealed hypocalcemia with a calcium level of 5.48 mg/dL (NV 9.4-10.8 mg/dl). Further examinations were conducted, showing a low ionized calcium level of 4.15 mg/dL (4.8-5.3 mg/dl), phosphorus of 6.1 mg/dL (3.5-6 mg/dL), PTH of 859 pg/mL (10-65 pg/ml), alkaline phosphatase of 181 UI/L (150-600 UI/L), and vitamin D (25 OH cholecalciferol) of 17 ng/mL (25-85 ng/ml). The conclusion from these results was a possible diagnosis of pseudohypoparathyroidism.

#### 2.1.1 Family and perinatal history

There was no consanguinity or relevant family history reported. Dichorionic diamniotic opposite-sex twin pregnancy was ended by Cesarean section due to the malposition of his twin. The full-term neonates were born at 37 + 5 weeks of gestation. The patient was born small for his gestational age with a birth weight of 2.250 g (-2.0 SD), birth length of 45 cm (-2.4 SD), and a head circumference of 31.5 cm (-1.46 SD). The birth weight of the female twin was normal at 2.700 g (-0.6 SD) with a length of 46 cm (SD –1.1 SD) and her head circumference was 32 cm (-0.9 SD). Both twins had a normal perinatal evolution.

#### 2.1.2 Past medical history

The patient had a first episode of seizure at 2 years and 10 months. In that context, he was febrile, concluding a diagnosis of atypical febrile seizure due to the seizure’s long duration, his slow recovery, and residual Todd´s paralysis on the left side of his body, which evolved successfully. Brain computed tomography (CT) and magnetic resonance imaging (MRI) were performed with normal results. However, electroencephalography showed epileptiform paroxysms in the occipital regions with right-sided predominance, and treatment with levetiracetam was started. The patient also had a history of mild congenital heart disease due to a bicuspid aortic valve and mild aortic stenosis, which is currently followed by the pediatric cardiology department with no current treatment.

#### 2.1.3 Physical examination

No apparent dysmorphia was observed. His current weight is 20 kg (-0.86 SD), with a height of 109 cm (0.99 SD). Tanner Stage 1 was assessed for both testes in the scrotum.

#### 2.1.4 Additional examinations

Blood test: glucose = 152 mg/dL, urea = 24.79 mg/dL, creatinine = 0.73 mg/dL, sodium = 135 mEq/L, potassium = 3.6 mEq/L, chloride = 103 mEq/L, total calcium = 5.48 mg/dL, ionized calcium = 4.15 mg/dL, phosphorus = 6.1 mg/dL, magnesium = 0.91 mg/dL, albumin = 4.02 g/dL, PTH = 859 pg/mL, alkaline phosphatase = 181 U/L, vitamin D (25 OH) = 17 ng/mL, AST = 38 U/L, and ALT = 12 U/L.

Urine test: creatinine = 34.79 mg/dL, calcium = 5.07 mg/dL, Ca/Cre ratio= 0.14 (normal values < 0.2), and phosphorus = 19.40 mg/dL. Complete blood count: 23,750 leukocytes/mm³ (32% L, 58% N, 0% C). Normal thyroid function. Nasopharyngeal swab: positive PCR for influenza A.

Normal chest X-ray.

#### 2.1.5 Evolution

Upon arrival at the emergency department, the patient presented with a generalized seizure and a temperature of 39°C. Antipyretics and intravenous midazolam (0.1 mg/kg) were administered, resolving the seizure.

Hypocalcemia was corrected with intravenous calcium infusion, followed by oral supplementation, with progressive correction of calcium levels, while maintaining phosphorus and magnesium within normal ranges.

Given the clinical suspicion of pseudohypoparathyroidism, treatment with calcitriol [1,25(OH)_2_ VitD]) 0.25 mcg/24 h, calcium carbonate 500 mg/12 h, and calcidiol (25OH VitD) 4000 IU/24 h was prescribed.

Follow-up of the patient was satisfactory, with normalization of the phosphate-calcium metabolism parameters after 2 months of the treatment (calcium 9.3 mg/dl, ionized calcium 5.07 mg/dl, phosphate 5.38 mg/dl, PTH 96 pg/ml, alkaline phosphatase 151 U/L, and 25OH vitamin D 42 ng/mL). Neurologically, while on the treatment with levetiracetam, the patient has not presented with new seizures, but a current electroencephalogram (EEG) (at the age of 4 years) continues to show the presence of epileptiform discharges.

### 2.2 Nucleic acid extraction

The genomic DNA of the patient and parents was extracted from peripheral blood using the QIAamp DNA Mini Kit (QIAGEN, Hilden, Germany), following the manufacturer’s instructions.

### 2.3 Molecular studies

#### 2.3.1 Methylation-specific multiplex ligation-dependent probe amplification

Dosage and methylation analyses for chromosome 20 were carried out using two different methylation-specific multiplex ligation-dependent probe amplification (MS-MLPA) experiments, following the manufacturer’s recommendations (MRC-Holland, Amsterdam, The Netherlands). For the study of the *GNAS* locus, the ME031-C1 kit was used. A custom MS-MLPA was designed to detect upd(20q)pat, including probes in the *MCTS2P*:TSS-DMR (20q11.21), *NNAT:* TSS-DMR (20q11.2-q12), *L3MBTL1*:alt-TSS-DMR (20q13.12), and *GNAS A/B*:TSS-DMR (20q13.32) (**Table 1**). These custom-designed probes were combined with the SALSA MLPA Probemix P200 Reference-1 kit, which contains reference probes for unique human DNA sequences and specific quality control fragments.

**Table 1:** Probe set of the custom-designed MS-MLPA kit (MS-MLPA UPD20q).

Probe name	*Locus*	Size (bp)	Probe sequence	
*MCTS2P*:TSS-DMR	20q11.21	110	L	GGGTTCCCTAAGGGTTGGAGACGTGGCGTTCAGAGAGCGGAAGTTGTCAGAT
			R	TTCACCGGGCCGTAAAGCGCGCTGGCTGTGTCATCTCTAGATTGGATCTTGCTGGCAC
*NNAT:*TSS-DMR	20q11.23	142	L	GGGTTCCCTAAGGGTTGGACCCTGCCCATTCCCTGCGCCGTCCTCCTCGCGCTGACCCTCCCTAGT
			R	GCGCCCGCGCCTGCCAGGGAACAAAGACTCGGGGCGCGGCGGGCGACCGCTGCTCTAGATTGGATCTTGCTGGCAC
*L3MBTL1*:alt-TSS-DMR	20q13.12	102	L	GGGTTCCCTAAGGGTTGGAGAGTCCTCAGACCCTCCCGCGCTCCAGT
			R	TCCGGATAAGCGAGTATAAGCCGCTGAACATGTCTAGATTGGATCTTGCTGGCAC
GNAS_A/B_03882-L22603_402*	20q13.32	126	L	GGGTTCCCTAAGGGTTGGAgtacaagaatctgaaCCGGCCGGCAGGCGCTGCCTTGCGTGT
			R	GAGTGCACCTCACTCACATGTAAGTCGGGGAGCGCactgaagTCTAGATTGGATCTTGCTGGCAC
GNAS_A/B_06191-L23094_238*	20q13.32	114	L	GGGTTCCCTAAGGGTTGGAgactgaCCAGTGCGTCCCCGGTGGGCCGATTTT
			R	TCGCGCTTCCCCTTCGGTTTATAGGGGCCGCTGCagttcTCTAGATTGGATCTTGCTGGCAC
GNAS_E13_03887-L03335_283*	20q13.32	106	L	GGGTTCCCTAAGGGTTGGAGAGGATCAGCACTGCCAGTGGAGA
			R	TGGGCGTCACTACTGCTACCCTCATTTCACCTGCGCTGTGTCTAGATTGGATCTTGCTGGCAC

The chromosomal band in which the probe is located, the total size of the probe, and its sequence are indicated (the sequences of the tails of the universal primers have been omitted). Lowercase residues refer to the stuffer sequence. L refers to the oligonucleotide on the left and R to the oligonucleotide on the right. The design of the probes marked with an asterisk (*) is from the ME031-C1-GNAS kit from MRC Holland.

The size of the fragments was evaluated with an ABI 3500 Genetic analyzer (Applied Biosystems, Foster City, California, USA), according to the manufacturer’s protocols. Data were analyzed using Coffalyser.net version 9.4 software (MRC-Holland).

#### 2.3.2 SNP array

The SNP array technique was used to confirm and determine the possible extent of the obtained findings by MS-MLPA. For this purpose, the CytoScan High-Density assay (ThermoFisher Scientific) was performed to genotype 906,600 SNPs, and the subsequent analysis was carried out using the recommended Chromosome Analysis Suite (ChAS) software version 4.5 (ThermoFisher Scientific).

#### 2.3.3 Microsatellite study

**Figure 1:**
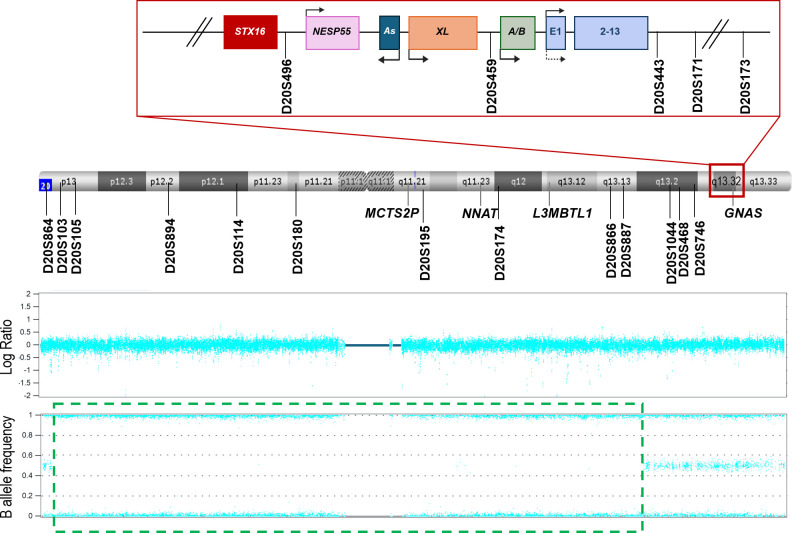
Schematic representation of chromosome 20, the distribution of the microsatellites studied, and the results obtained from the SNP array. The *GNAS* locus (red square) has been magnified to view it in more detail. The different DMRs of the locus are denoted by pink (*NESP55*), dark blue (AS), orange (XL), and green (A/B) squares. The regulator gene *STX16* (upstream *GNAS* locus) is represented by a red square. The *GNAS* gene is drawn in light blue. Arrows indicate the transcription sense. *MCTS2P, NNAT*, and *L3MBTL1* are other imprinted loci in the chromosome. The position of each STR analyzed throughout the chromosome and in the *GNAS* locus is indicated with a straight line. In the lower part of the image, the SNP array results are presented. In the Log Ratio panel, no copy number variations are shown. In the lower panel, the green dashed square delimits a zone with no heterozygosity.

The STR study was performed for the trio by fluorescent polymerase chain reaction (PCR), using different markers along the long arm (*D20S496, D20S459, D20S443, D20S171, D20S173, D20S1044, D20S468, D20S746, D20S195, D20S174, D20S866*, and *D20S891*) and short arm (*D20S864, D20S103, D20S105, D20S894, D20S114* and *D20S180*) of chromosome 20 (**Figure 1**).

The thermocycling program was: 95°C (5 min); 35x{95°C (30 s); 55°C (30 s); 72°C (30 s)}; 72°C (7 min); 60°C (1 h); 4°C (∞). After thermocycling, the product was visualized in an agarose gel (1.5% w/v) electrophoresis with 1x TAE (Thermo Fisher Scientific, Waltham, Massachusetts, USA) and subsequent dilutions were performed.

The fragments’ size was determined with an ABI 3500 Genetic analyzer. The results were analyzed with GeneMapper Software 5 (Applied Biosystems).

## 3 Results

### 3.1 *GNAS* MS-MLPA

The methylation status of the *GNAS* locus was evaluated and complete methylation alteration was found in all the DMRs (Data not shown). Specifically, gain of methylation (GOM) was present in the *GNAS-NESP:* TSS-DMR and LOM in the *GNAS-AS1*:TSS-DMR, *GNAS-XL:*Ex1-DMR and *GNAS A/B*:TSS-DMR. We also detected that no copy number variations were present. As the methylation was completely altered, a paternal UPD needed to be checked.

### 3.2 SNP array

To confirm/discard UPD, a SNP array in the proband case was performed, revealing loss of heterozygosity from 20p13 to 20q13.13 (**Figure 1**), suggesting either the possibility of homozygosity due to a high level of consanguinity or an isodisomy in that zone (not including *GNAS*).

No other regions with loss of heterozygosity were detected.

### 3.3 Custom upd(20q)pat MS-MLPA

**Figure 2:**
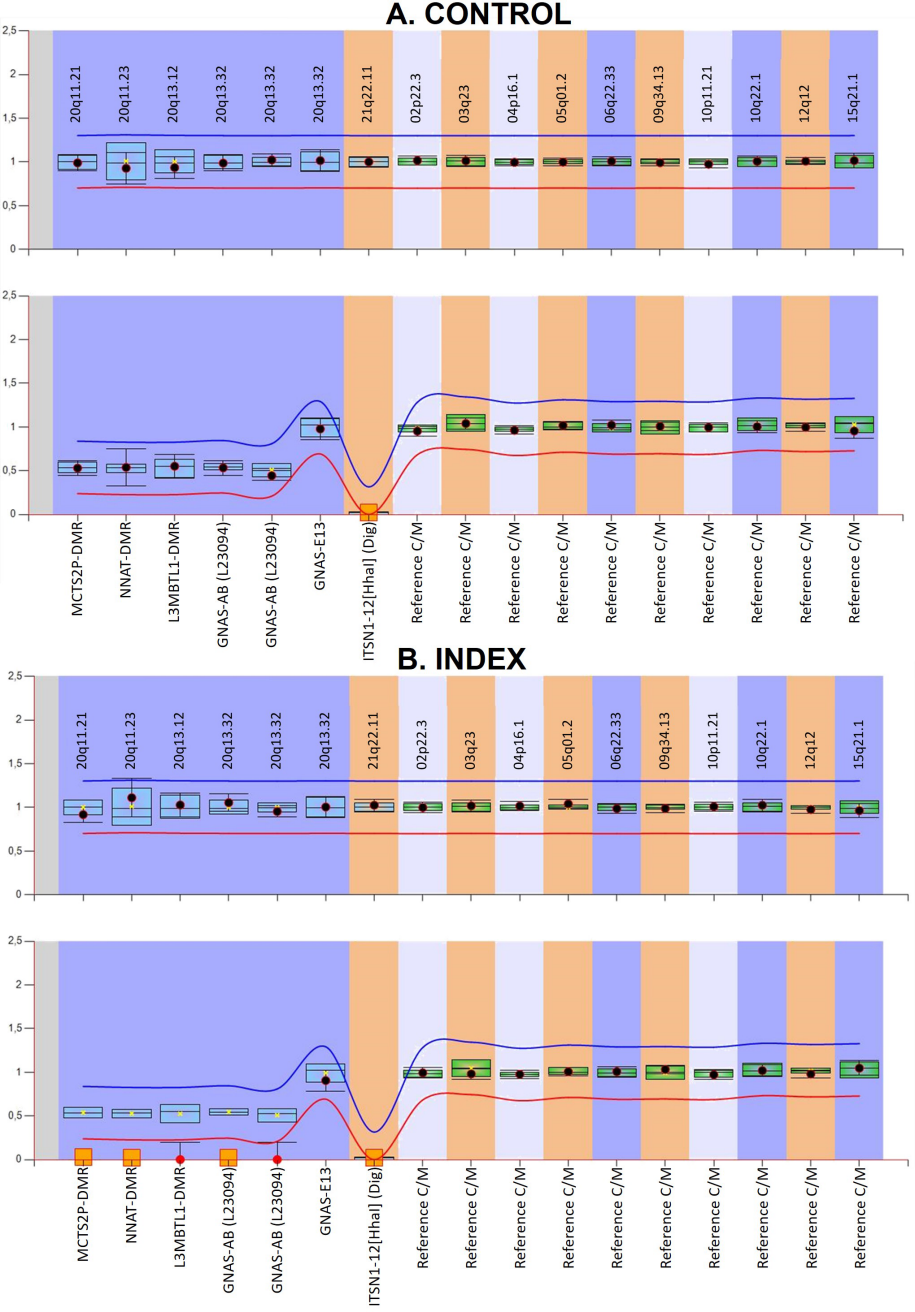
Graphical representation of the custom MS-MLPA results viewed with Coffalyser analysis software. **(A)** shows the results of a control and **(B)** corresponds to the index case presenting with iPPSD3. In the upper panel for each individual, the genomic dosage is shown. In the lower panels, the methylation status is represented. In the methylation graphic, the imprinted DMRs should be 0.5, while in biallelically expressed ones, the result should be 1 if they do not have the *HhaI* digestion site. In contrast, digestion control probes must show a 0. The obtained from the reference samples for each probe is represented by the blue (if on chromosome 20) or green (if on other genomic regions) bars and the 95% confidence interval for the sample value under study is represented by the point error bars. The mean values of five control subjects were used for the assessment of relative copy number and methylation percentage. The red and blue lines represent the arbitrary limits that are within ±0.3 of the mean value of each probe in the reference samples. Loss-of-methylation is represented by red circles and orange squares.

The custom MS-MLPA for detecting UPD in chromosome 20q gave the results represented in **Figure 2**. Briefly, it can be observed that the *GNAS* locus and the DMRs upstream of *GNAS* present a complete LOM in the patient, suggesting that the observed loss of heterozygosity could be the consequence of an upd(20q)pat. The digestion control probes and the reference probes showed the expected methylation and genomic levels, indicating that the experiment worked well. A control individual with no disease was included, and, as expected, no methylation alterations or copy number variants (CNVs) were detected.

### 3.4 Microsatellite study

Later on, access to parental samples was possible, so, to confirm the results obtained from the SNP array and custom upd(20q) MS-MLPA, different STRs throughout the *GNAS* locus, 20p, and 20q regions were studied. The distribution of the microsatellites along the chromosome is depicted in **Figure 1** and the results obtained are summarized in **Table 2**.

**Table 2:** Genotypes obtained for the microsatellites studied in chromosome 20.

STR	INDEX	MOTHER	FATHER
Short arm (20p)			
*D20S864*	225/225	225/229	225/225
*D20S103*	97/99	95/99	97/99
*D20S105*	237/245	237/237	237/245
*D20S894*	265/265	257/271	265/269
*D20S114*	260/260	262/262	260/260
*D20S180*	149/149	149/149	149/149
Long arm (20q)			
*D20S195*	240/240	246/256	240/240
*D20S174*	298/298	278/294	298/298
*D20S866*	169/169	165/175	169/177
*D20S887*	243/243	249/251	243/243
*D20S1044*	207/207	207/207	207/207
*D20S468*	204/204	188/198	204/204
*D20S746*	249/249	249/249	249/249
*D20S496*	203/203	203/203	203/203
*D20S459*	226/226	226/226	226/226
*D20S443*	136/142	142/142	136/142
*D20S171*	129/137	131/133	129/137
*D20S173*	179/187	179/179	179/187

The genotype of the three members of the family is presented for the different STRs (short tandem repeats / microsatellites). Light grey indicates the isodisomic zone, while dark grey marks the heterodisomic zone. Underlined are the highlighted informative results.

As it can be seen in **Table 2**, the results confirmed that the patient presents a mixed upd(20q)pat, with an isodisomic region that does not include *GNAS* and a heterodisomic zone in both telomeric regions.

## 4 Discussion

*GNAS* is a complex locus characterized by a specific imprinting pattern. When the methylation is altered, at least when hypomethylation in the *GNAS A/B:*TSS-DMR happens, patients develop iPPSD3 (10). The patient described in this work presented with a complete methylation defect affecting all four DMRs detected by the MS-MLPA specific for the *GNAS* locus, so he was classified as a sporadic case. In patients with complete alteration, paternal UPD should be tested, specifically, isodisomy of chromosome 20 [iUPD (20)] affecting the chromosomic region that includes the *GNAS* locus (10, 21–23).

Following the current guidelines (10), the genetic material of the proband was analyzed with an SNP array. An SNP array is a good technique to detect iUPD since in cases where a single homolog chromosome is transmitted in duplicate from a parent, whole (or a large part of) chromosomal homozygosity can be seen (23). As is shown in **Figure 1**, there is a central zone with no heterozygosity in the patient. There are two possible reasons for this. First, when blocks of homozygosity are present in different chromosomes, parental consanguinity is suggested (24). However, at the genetic counseling consultation, the parents denied any relatedness and the SNP array results only showed loss-of-heterozygosity (LOH) in chromosome 20. Second, if large blocks of homozygosity are restricted to a single chromosome, it could represent UPD (25). Still, it was surprising that in the SNP array, there was a region with no LOH in which the *GNAS* locus was included. This could be a consequence of a chromosome with both isodisomy and heterodisomy. To confirm this, a custom MS-MLPA was designed and performed, confirming the loss of methylation at the four maternal DMRs located at 20q [*MCTS2P*:TSS-DMR (20q11.21), *NNAT:*TSS-DMR (20q11.2-q12), *L3MBTL1*:alt-TSS-DMR (20q13.12), and *GNAS A/B:*TSS-DMR (20q13.32)], which supported the possibility of UPD and was suggestive of a mixed disomy throughout chromosome 20.

Fortunately, to confirm this, samples from the parents were finally available and STR analysis was performed, with the STRs located around chromosome 20. Thus, as shown in **Figure 1**; **Table 2**, the patient under study shows a combination of hetero- and isodisomy of chromosome 20. We would like to draw attention to this study of STRs. If, instead of the SNP array, we had initially analyzed the markers flanking *GNAS* (*D20S496, D20S459, D20S443, D20S171, D20S173*) in the absence of parental sample (as was in this situation), we would have ruled out iUPD as the patient carried a heterozygous genotype for some of them. And, since hUPD had been demonstrated once by STRs and the SNP array for the *GNAS* locus (17), this patient would have been characterized as spor-PHP1B/iPPSD3 with no underlying genetic cause. The correct (epi)genetic diagnosis of this disease allows for appropriate genetic counseling, as it is possible to establish the risk of recurrence and transmission of the disease in the presence of an underlying genetic alteration, whereas in the case of epimutation it would, for the time being, be unknown. Furthermore, if the patient carries iUPD, additional clinical manifestations secondary to an unmasked recessive disease may arise.

Moreover, from a research perspective, the correct subclassification of the different types of iPPSD3 cohorts [according to their (epi)genotype] could help to define clinical-epigenetic relationships and improve prognostic information. Thus, in the absence of parental samples, complete methylation defects at *GNAS* cannot be determined if it is due to an hUPD (20, 26), and, even for iUPD detection, a SNP array can be more informative than STRs (23), both have their limitations for hUPD identification. As an alternative, we propose the use of an MS-MLPA to examine additional DMRs at this chromosomal arm, as in our case.

It has already been well-demonstrated in the literature that this combination, called mixed UPD, is a molecular disease-causing mechanism described for several chromosomes (25, 27, 28). Regarding iPPSD3, researchers have also detected this genetic defect in chromosome 20, with an interstitial isodisomy in 20q affecting *GNAS* (29). Mixed UPD occurs when a chromosome contains both isodisomic and heterodisomic parts as a consequence of meiotic nondisjunction events. If the error occurs during meiosis I, a zone of homozygosity will be seen in the terminal region of the chromosomes. However, if the error is in meiosis II, the region of homozygosity is present in the centromeric region (23).

UPDs occur as a consequence of at least two independent errors during meiosis or after fertilization, so they do not follow a Mendelian inheritance (29) and are not necessarily disease-causing because no genetic material is lost or gained. However, some specific events lead to disease, as is the case in imprinting. The chromosomes that contain imprinted loci, as is the case for chromosome 20 and the *GNAS* locus, are sensitive to UPD. This happens because, due to UPD, the balance of methylation that leads to parental-dependent transcription is altered (23, 25).

In our study, we describe a patient with iPPSD3 with an heterodisomy in the *GNAS* locus. This is novel since even though iUPD(20q)pat is widely described, it is rare to find an heterodisomy as the cause of iPPSD3. In fact, only one case is described in the literature, and, as in our case, it was caused by a mixed UPD, and *GNAS* was found in the heterodisomic zone (17). In the reported patient, there were no informative STRs nor SNPs in the *GNAS* locus. There was one informative STR in the *STX16* gene. The mutation rate of STR must be taken into account, which is quite a common event (30, 31) and could lead to a difficulty in heterodisomy confirmation. To solve this problem, we propose the inclusion of a novel custom-designed MS-MLPA to detect upd(20q). This custom-designed MS-MLPA includes probes that hybridize in different DMRs along the long arm of chromosome 20: *MCTS2P*:TSS-DMR, *NNAT:* TSS-DMR and *L3MBTL1*:alt-TSS-DMR. Thanks to the imprinted nature of these DMRs (32–34), it has been possible to detect an alteration in the methylation status (**Figure 2**), showing complete hypomethylation. The combined information from the STRs/SNP array and this novel assay allowed us to confirm the mixed upd (20).

Although it is extremely rare, maternal UPD in chromosome 20 (upd(20)mat) has been detected in some patients. This genetic condition causes a poorly characterized syndrome known as Mulchandani-Bhoj-Conlin (MBCS, OMIM#617352). The main characteristics of this syndrome are growth failure (intrauterine/postnatal growth retardation) and feeding difficulties (35, 36). As the cause of MBCS is an UPD in chromosome 20, the MS-MLPA developed for the diagnosis of iPPSD3 can also be used for the detection of upd(20)mat and proceed to its diagnosis. At the moment, the specific (epi)genetic cause of MBCS still remains unknown, thus, a limitation of this custom MS-MLPA for the diagnosis of MBCS is that only 20q has been studied.

In conclusion, if the study of STRs is only done in the index case and it shows an heterozygote genotype, it is not enough to discard an UPD, as we present an extra confirmation that hUPD is possible at this locus. In this work, we have also presented a custom MS-MLPA for DMRs in the long arm of chromosome 20. Its efficiency in detecting upd(20q)pat as the underlying genetic cause in iPPSD3 cases has been demonstrated.

## Data Availability

The original contributions presented in the study are included in the article, further inquiries can be directed to the corresponding author.
